# Pharmacological effects and the related mechanism of scutellarin on inflammation-related diseases: a review

**DOI:** 10.3389/fphar.2024.1463140

**Published:** 2024-08-12

**Authors:** Yang Zhou, Chenlin Gu, Yan Zhu, Yuting Zhu, Yutong Chen, Li Shi, Yang Yang, Xin Lu, Hanqing Pang

**Affiliations:** ^1^ School of Medicine, Institute of Translational Medicine, Yangzhou University, Yangzhou, China; ^2^ Jiangsu Key Laboratory of Integrated Traditional Chinese and Western Medicine for Prevention and Treatment of Senile Diseases, Yangzhou University, Yangzhou, China

**Keywords:** scutellarin, inflammation, NF-κB, MAPK, PI3K/AKT, Nrf2/ARE

## Abstract

Inflammation is a biological response of multicellular organisms caused by injuries, pathogens or irritants. An excessive inflammatory response can lead to tissue damage and various chronic diseases. Chronic inflammation is a common feature of many diseases, making the search for drugs to treat inflammation-related diseases urgent. Scutellarin, a natural flavonoid metabolite, is widely used in the treatment of various inflammation-related diseases for its anti-inflammatory, anti-oxidant and anti-cancer activities. Scutellarin can inhibit key inflammatory pathways (PI3K/Akt, MAPK, and NF-κB, etc.) and activate the anti-oxidant related pathways (Nrf2, ARE, ect.), thereby protecting tissues from inflammation and oxidative stress. Modern extraction technologies, such as microwave-assisted, ultrasound assisted, and supercritical fluid extraction, have been utilized to extract scutellarin from Scutellaria and Erigeron genera. These technologies improve efficiency and retain biological activity, making scutellarin suitable for large-scale production. Scutellarin has significant therapeutic effects in treating osteoarthritis, pulmonary fibrosis, kidney injury, and cardiovascular diseases. However, due to its low bioavailability and short half-life, its clinical application is limited. Researchers are exploring innovative formulations (β-cyclodextrin polymers, triglyceride mimetic active ingredients, and liposome precursors, etc.) to improve stability and absorption rates. Despite these challenges, the potential of scutellarin in anti-inflammatory, anti-oxidant, and anti-cancer applications remains enormous. By optimizing formulations, exploring combination therapies, and conducting in-depth mechanistic research, scutellarin can play an important role in treating various inflammatory diseases, providing patients with more and effective treatment options.

## 1 Introduction

Inflammation is a biological response of multicellular organism caused by sensitizing stimuli including damaged cells, pathogens or irritants. Moderate inflammatory response plays a crucial role in our long-term health through clearing infections and repairing tissue damage (Goodman et al., 2019; Henein et al., 2022; Soliman and Barreda, 2022). While over-activation of inflammation could lead to tissue damage and various chronic diseases. Chronic inflammation is a well-recognized feature of most diseases, dramatically increasing global morbidity and mortality (Xia et al., 2023). Osteoarthritis (OA) is the most universal inflammatory disease of the joints (about 1 billion people), which brought an economic burden of nearly $140 billion (Wang et al., 2024). It is estimated that the majority of people suffer from OA by the age of 40–50 years and the incidence of OA is increasing due to the aging population and the high incidence rate of obesity (Jiang et al., 2024). Fibrosis, resulting from impaired tissue repair and remodeling, is a common long-term outcome of chronic inflammatory diseases (Massoud et al., 2024). Pulmonary fibrosis is characterized by fibroproliferation and abnormal tissue repair after injury, which could exacerbate the pathological process of pulmonary fibrosis through oxidative stress and inflammation-induced immune responses (Li Z. et al., 2024). In addition, inflammation is an important cause of excessive cardiovascular risk and progressive kidney injury. Chronic kidney disease accelerated dysregulation of the innate and adaptive immune systems, which would make the systemic inflammation and localized vascular inflammatory responses increase continuously, ultimately lead to atherosclerosis and microcirculatory dysfunction (Huck et al., 2024). That’s why the research for drugs to treat inflammation-related diseases is so urgent.

Scutellarin, a natural flavonoid isolated from the root of *Scutellaria baicalensis* Georgi [Lamiaceae; Scutellaria], has been extensively used in the treatment of various diseases for its valuable biological activities, such as anti-oxidant, anti-inflammatory, and anti-cancer activities (Bian et al., 2020; Song et al., 2020). The mechanism by which scutellarin prevented and treated inflammation-related diseases included inhibiting the production of inflammatory mediators or oxidative stress responses. Cytokines and chemokines mediate the recruitment, activation, and proliferation of neutrophils, and promote the extravasation of monocytes and macrophages to the site of inflammation. These pro-inflammatory cytokines (IL-1, IL-6 and TNF-α, etc.) can activate the inflammatory cascades, thereby exacerbating organ and tissue injuries (Duan et al., 2024). Researches have shown that scutellarin can inhibit the production of these cytokines and reduce inflammatory responses (Cao et al., 2019; Aksit et al., 2023). For example, NF-κB is a key transcription factor that coordinates the expression of multiple genes associated with inflammatory responses (Mussbacher et al., 2023). Scutellarin can reduce the secretion of inflammatory mediators and significantly regulate the production of immune effector factors by mediating the NF-κB signaling pathway (Zhang et al., 2022). In addition, oxidative stress is another factor that exacerbates tissue damage caused by inflammatory reactions. The reactive oxygen species (ROS) produced during oxidative stress can activate various inflammatory signaling pathways, thereby promoting inflammation cascades. Scutellarin has been reported to have anti-oxidant properties, which can clear ROS, thereby reducing oxidative stress and reversing the trend towards inflammation (Zhang et al., 2022).

Scutellarin may have significant advantages in the treatment of inflammation-related diseases. Nevertheless, the low bioavailability and short half-life of flavonoids have limited the therapeutic efficacy of scutellarin in inflammation-related diseases. To solve the problem, researchers are committed to exploring new formulations of flavonoids including polymeric nanoparticles, liposomes, solid lipid microparticles, nanogels and nanocrystals (Sharma and Wairkar, 2024). However, whether the new formulations of scutellarin are economical, safe and effective is yet to be investigated, and the direction of specific drug dosage forms and delivery modalities of scutellarin still need to be explored in greater depth. Up to date, there has not been a systematic and complete review of the specific mechanisms of scutellarin against inflammation-related diseases, which has hindered its application and development as a good anti-inflammatory agent. This review is to provide valuable information for future applications of scutellarin in preventing and treating inflammation-related diseases.

## 2 Main sources and extraction methods of scutellarin

**FIGURE 1:**
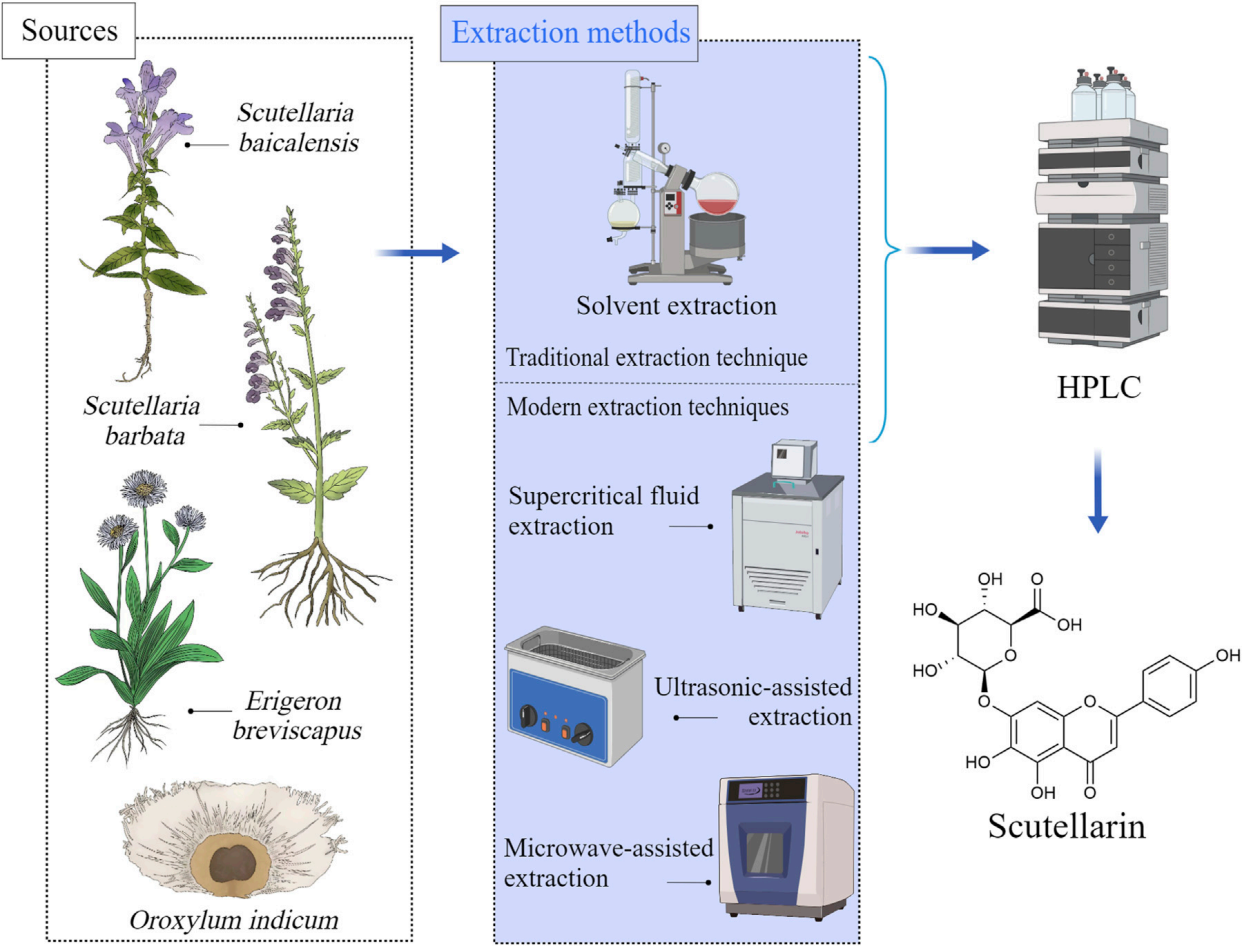
Sources of scutellarin and its available extraction methods, Created with MedPeer (medpeer.cn).

Scutellarin is a flavonoid glycoside mainly present in Scutellaria and Erigerontis genera (Chledzik et al., 2018). Scutellarin is frequently existed in *S. baicalensis* (Pei et al., 2022), and it was also identified in *Scutellaria lateriflora* L. [Lamiaceae; Scutellaria], *Scutellaria barbata* D.Don [Lamiaceae; Scutellaria], and *Oroxylum indicum* (L.) Kurz [Bignoniaceae; Oroxylum] as well. The Scutellaria genus is widely distributed in east Asia and north America (Irvin et al., 2019). The roots, stems and leaves of these plants contain large amounts of scutellarin. Breviscapine, the extract of *Erigeron brevisacapus* (Vaniot) Hand.-Mazz. [Compositae; Erigeron] that is rich in scutellarin, has been reported to be effective for improving blood circulation. Studies revealed that the contents of scutellarin varied significantly depending on the plant species and parts (Georgieva et al., 2020). For example, the roots of *S. baicalensis* and the whole herb of *E. brevisacapus* are the main resources for extracting scutellarin (Nurul Islam et al., 2011; Georgieva et al., 2022). The sources of scutellarin and its available extraction methods were shown in Figure 1.

The extraction methods of scutellarin included traditional extraction techniques and modern extraction techniques (Hao et al., 2020). For traditional extraction methods (solvent extraction and hot reflux extraction), various solvents such as ethanol, methanol, or water can be used to extract scutellarin from plant materials (Yang et al., 2019b). Although these methods are easy to operate and have low equipment costs, they have disadvantages such as low efficiency and long-time consumption. With the development of science and technology, modern extraction techniques have greatly improved the extraction efficiency and purity of scutellarin (Xia et al., 2021). Ultrasound assisted extraction (UAE) utilizes the cavitation effect of ultrasound to accelerate the contact between solvents and plant materials, which could improve extraction efficiency, reduce solvent usage, and achieve an extraction rate of 50%–60% for scutellarin (Xiang and Wu, 2017). Microwave assisted extraction (MAE) utilizes microwave energy to rapidly heat solvents and plant materials, rapidly damaging cell walls and releasing scutellarin. The extraction rate of scutellarin is very high (about 70%–80%) (Li et al., 2015). Supercritical fluid extraction (SFE) uses supercritical carbon dioxide as a solvent under high pressure to preserve the biological activity of scutellarin, with an extraction rate of approximately 60%–70% (Yang and Wei, 2018; Yang et al., 2019a). These modern technologies not only have significant advantages in extraction efficiency, but also maintain the biological activity of scutellarin, making them suitable for large-scale industrial production and high-value extraction. The advantages and disadvantages of scutellarin extraction methods were summarized in Table 1.

**TABLE 1:** Principles and advantages and disadvantages of scutellarin extraction methods.

Extraction	Methods Basic	Advantages	Disadvantages	Refs
Solvent Extraction	Solvent is used to dissolve scutellarin in the plant material and the active metabolite is extracted through an infusion process	Simple operation and low equipment requirement	Extraction efficiency is low and time consuming	Yang et al. (2019b)
Ultrasonic-assisted extraction	Utilizing the cavitation effect of ultrasonic waves to accelerate the contact between the plant material and the solvent and improve the extraction efficiency	Short time and high extraction efficiency	Specialized ultrasonic extraction equipment is required	Xiang and Wu (2017)
Microwave-assisted extraction	Utilizes microwave energy to heat the solvent and plant material to promote the release and dissolution of active metabolites	High extraction speed and low energy consumption	Requires specialized microwave extraction equipment	Li et al. (2015)
Supercritical fluid extraction	Extraction of Scutellarin from plant materials under high pressure using supercritical CO_2_ as a solvent	Highly efficient and environmentally friendly, able to maintain the biological activity of Scutellarin	Expensive equipment and complicated operation	Yang and Wei (2018), Yang et al. (2019a)

## 3 Role of scutellarin on inflammatory signaling pathways

### 3.1 Inhibition of NF-κB pathways

#### 3.1.1 Inhibition of IKK activation and IκBα phosphorylation

Under normal circumstances, nuclear factor-kappa B (NF-κB) was present in the cytoplasm as an inactive state, which was combined with the IκB proteins (Barnabei et al., 2021). When inflammatory response (such as LPS, cytokines, etc.) was initiated (Yang et al., 2021), the IκB kinase (IKK) complex would be activated (Moser et al., 2021), leading to IκBα phosphorylation, which could further promote the release of NF-κB and the degradation of the IκBα (Shen et al., 2019). Scutellarin could inhibit the activated IKK, thereby preventing the degradation and phosphorylation of IκBα (Peng et al., 2020; Park et al., 2022). As a consequence, scutellarin could make NF-κB binding to IκBα in the cytoplasm, and the inactivated NF-κB cannot exert its effects (Lu et al., 2021; Xu et al., 2021).

#### 3.1.2 Prevention of NF-κB nuclear translocation

NF-κB translocation from the cytoplasm to the nucleus is necessary for inflammatory gene transcription. Scutellarin inhibited the nuclear translocation of the p65 subunit by stabilizing IκBα (Giridharan and Srinivasan, 2018). Studies indicated that the nuclear distribution of NF-κB p65 subunit was mainly present in the cytoplasm after scutellarin treatment (Wang et al., 2011; You et al., 2018). Scutellarin prevented nuclear translocation of NF-κB and thus inhibited its activity (Peng et al., 2020).

#### 3.1.3 Reduction of NF-κB target gene expression

**FIGURE 2:**
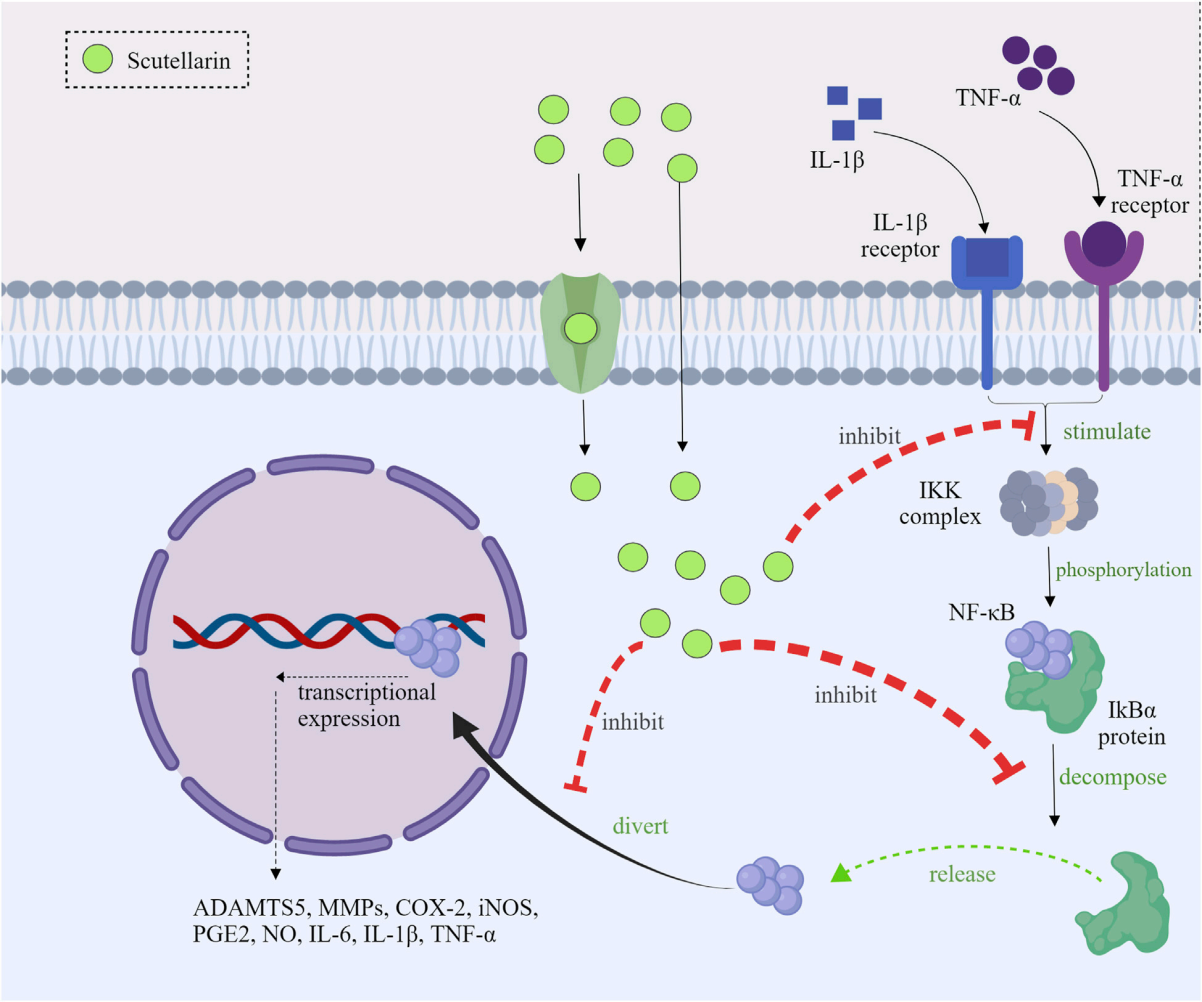
Mechanism diagram of scutellarin inhibiting the NF-κB pathways, Created with MedPeer (medpeer.cn).

After entering into the nucleus, NF-κB activated transcription of a set of inflammatory genes (Alharbi et al., 2021). Scutellarin suppressed the expression of these inflammatory genes to attenuate inflammation. It has been shown that scutellarin significantly reduced LPS-induced mRNA and protein levels of IL-1β, IL-6, TNF-α, COX-2, and iNOS (Peng et al., 2020; Park et al., 2022; Aksit et al., 2023; Zhang et al., 2023; Duan et al., 2024). Scutellarin could alleviate the local and systemic inflammatory response through decreasing the expression of those pro-inflammatory mediators *in vivo* (Figure 2).

### 3.2 Inhibition of MAPK pathways

The major isozymes of the mitogen-activated protein kinase (MAPK) family included p38, JNK and ERK (Guo et al., 2020; Iroegbu et al., 2021; Van Gerrewey and Chung, 2024), the front two are closely related to inflammation. Scutellarin can be used as a therapeutic agent to inhibit inflammation by modulating the MAPK pathways.

#### 3.2.1 Regulation of the JNK pathway

The JNK (c-Jun N-terminal kinase) signaling pathway is involved in cell stress response, apoptosis and inflammation. The JNK pathway is activated when cells are exposed to ultraviolet (UV) light, oxidative stress and cytokines (Liu B. et al., 2020). Upon activation, JNK can make the downstream transcription factor c-Jun phosphorylation, which plays a key role in gene expression associated with cell stress response or apoptosis. It has been demonstrated that dysregulated JNK signaling is closely related to numerous inflammatory diseases as well as cancers (Zhang et al., 2019). Scutellarin influenced the process of inflammation by regulating multiple factors of the JNK pathway. Scutellarin prevented JNK phosphorylation, thereby leading to the decrease in pro-inflammatory gene expression (You et al., 2018; Deng et al., 2024). Moreover, scutellarin also significantly reduced inflammation via inhibiting IL-1β and TNF-α production (You et al., 2018; Sun et al., 2019; Chen H. L. et al., 2020).

#### 3.2.2 Regulation of the p38 pathway

**FIGURE 3:**
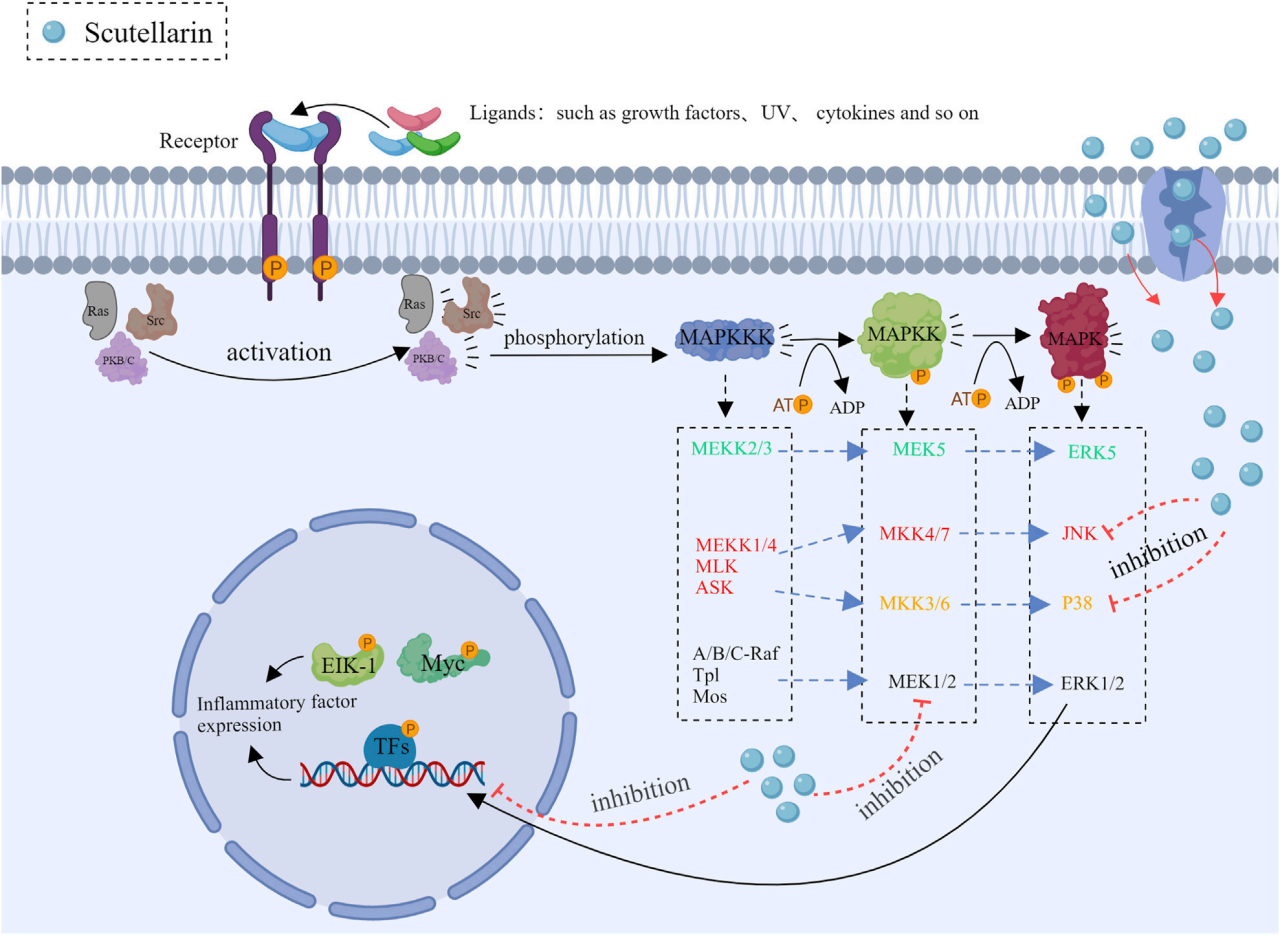
Mechanism diagram of scutellarin inhibiting the MAPK pathways, Created with MedPeer (medpeer.cn).

The p38 pathway plays a key role in cellular stress response and inflammation (Yong et al., 2009). It is activated by stress kinases such as MKK3 and MKK6 (Rossa et al., 2006), which phosphorylate and activate downstream effector proteins, thereby affecting the production of inflammatory factors, cell proliferation, differentiation, and apoptosis. Scutellarin regulates the p38 pathway through various specific mechanisms, thereby exerting its anti-inflammatory effects (You et al., 2018). Scutellarin can also reduce the phosphorylation levels of transcription factors ATF2 and CHOP, causing the low expression of inflammatory genes (Wang Z. et al., 2022). Together, these results demonstrated that scutellarin could exert the potent anti-inflammation effects through regulating p38 pathway (Figure 3).

### 3.3 Regulation of PI3K/Akt Pathway

**FIGURE 4:**
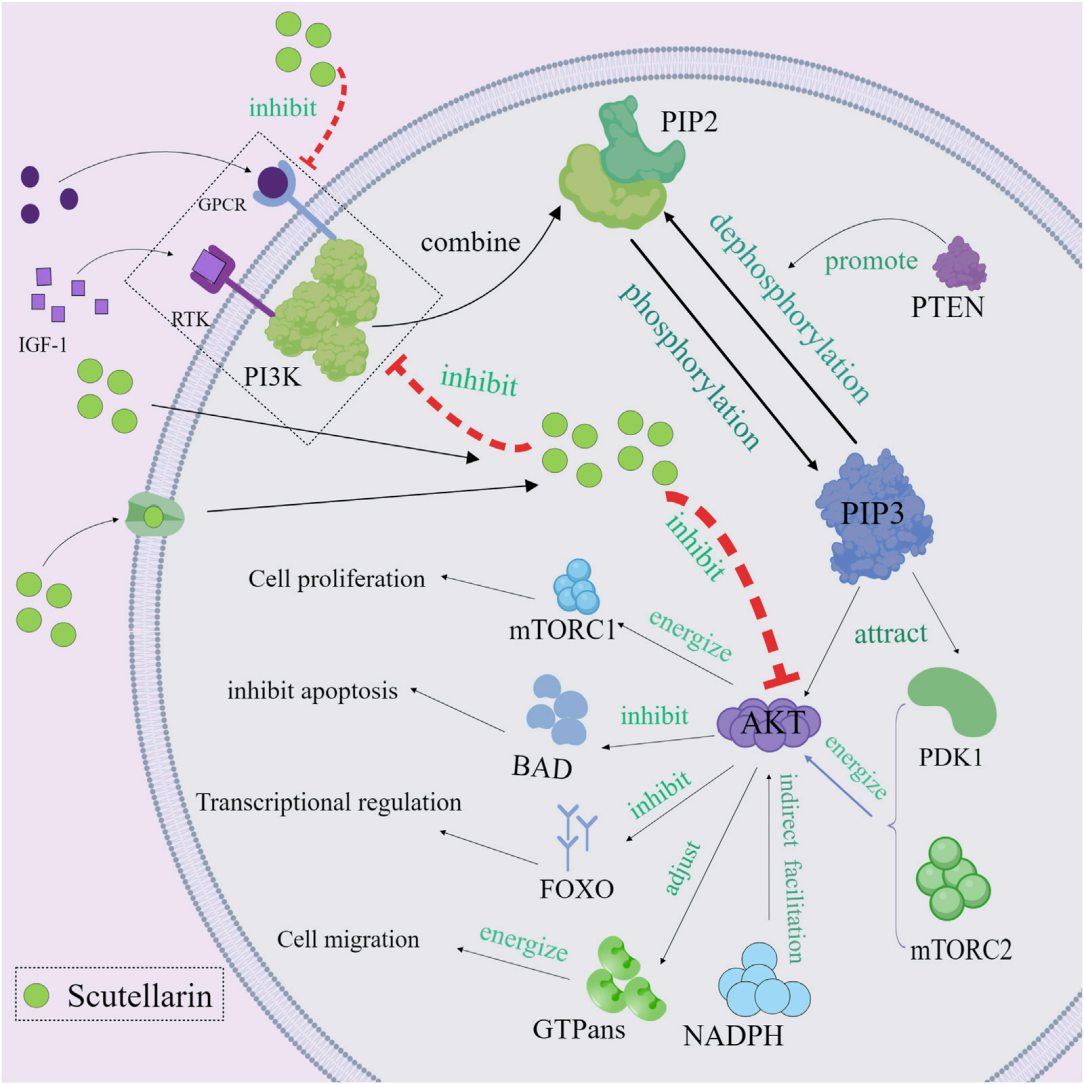
Mechanism diagram of scutellarin regulating of PI3K/Akt Pathway, Created with MedPeer (medpeer.cn).

The phosphatidylinositol 3-kinase/protein kinase B (PI3K/Akt) pathway plays a critical role in cell growth, survival, proliferation, and metabolism (Yong et al., 2009; Deng and Zhou, 2023). Aberrant activation of the PI3K/Akt pathway is strongly associated with cancer and inflammatory diseases (Alzahrani, 2019; Ghafouri-Fard et al., 2021). Scutellarin exerted the anti-inflammatory and anti-cancer effects through regulating the PI3K/Akt pathway (Wang et al., 2019; Hu et al., 2022). Mechanistically, receptor phosphorylation recruits and activates PI3K, which phosphorylates PIP2 to PIP3 (Carnero et al., 2008). The PH structural domain of Akt binds to PIP3, which recruits Akt to the cell membrane, thereby localizing Akt to the cell membrane and activating it. Akt is then phosphorylated and activated by pyruvate dehydrogenase kinase, isozyme 1 (PDK1) and mechanistic target of rapamycin complex 2 (mTORC2) (Xu et al., 2020). The fully activated Akt then enters the cytoplasm or nucleus to phosphorylate its downstream effector proteins. Scutellarin could inhibit PI3K activity, prevent phosphatidylinositol 4,5-bisphosphate 3 (PIP3) production, reduce Akt membrane localization, and directly inhibit Akt phosphorylation (Zhang et al., 2023). In this way, Akt could not be effectively phosphorylated, leading to the inactivated downstream effector proteins (mTOR, Bax, etc.) (Ju et al., 2021; Wang Z. et al., 2022). mTOR could promote the production of pro-inflammatory cytokines (such as IL-6 and TNF-α) by regulating translation factors 4E-BP1 and S6K. Therefore, inhibiting mTOR activity can reduce the expression of pro-inflammatory cytokines and alleviate the inflammatory response. Many current studies have discovered that scutellarin greatly reduced Akt-mediated pro-inflammatory cytokines such as TNF-α, IL-1β and IL-6, thereby attenuating the inflammatory response and tissue damage (Figure 4).

### 3.4 Activation of Nrf2/ARE pathway

**FIGURE 5:**
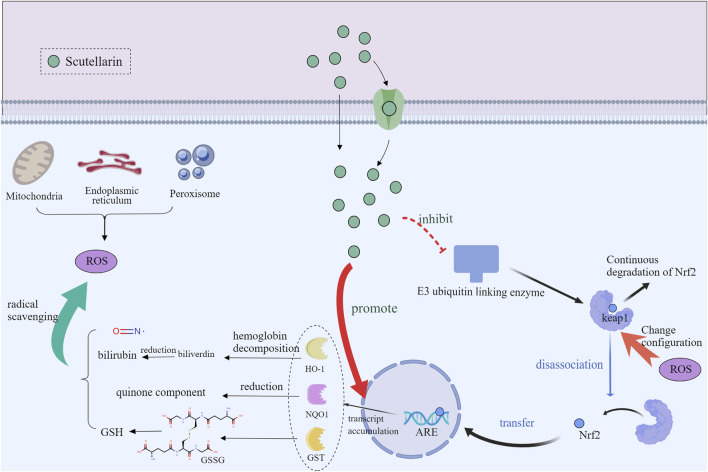
Mechanism diagram of scutellarin regulating of Nrf2/ARE Pathway, Created with MedPeer (medpeer.cn).

The nuclear factor E2-associated factor 2/anti-oxidant response element (Nrf2/ARE) pathway is a key regulatory mechanism for cellular anti-oxidant and detoxification responses (Zgorzynska et al., 2021). Normally, Nrf2 is inhibited by kelch-like ECH-associated protein 1 (Keap1) protein and localized in the cytoplasm (Panieri and Saso, 2021; Song et al., 2021). Under oxidative stress or electrophilic conditions, Nrf2 is released from Keap1 and translocated to the nucleus. It has been shown that scutellarin promoted the nuclear translocation and release of Nrf2 by interfering with the interaction between Nrf2 and Keap1 (Li J. et al., 2023; Shahmohammadi et al., 2023). After entering the nucleus, Nrf2 initiates transcription of anti-oxidant genes through binding to ARE. Activated Nrf2 regulates the expression of a range of anti-oxidant, detoxification and anti-inflammation enzymes, such as heme oxygenase-1 (HO-1) (Zhang et al., 2023) and superoxide dismutase (SOD) (Shahmohammadi et al., 2023; Xie et al., 2023). By triggering the Nrf2/ARE pathway, scutellarin increases the expression levels of these anti-oxidant enzymes, which enhances cellular anti-oxidant capacity and protects cells from oxidative stress toxic damage and inflammation (Figure 5).

## 4 Specific role of scutellarin in inflammation-related diseases

Due to the obvious anti-inflammation and anti-oxidative effects, scutellarin has been used to treat osteoarthritis, pulmonary fibrosis, kidney injury and cardiovascular diseases (Table 2).

**TABLE 2:** Mechanisms and modulation of scutellarin for the treatment of inflammation-related diseases.

Inflammation-related Diseases	Mechanism of action	Trends in action	Refs
Pulmonary fibrosis	NF-κB/NLRP3-mediated epithelial–mesenchymal transition and inflammation (−)	p-p65/p65, IκBα, NLRP3, CASP1, ASC, IL-1β, IL-18, GSDMDN, FN, VIM, CDH2, MMP-2, MMP-9↓	Peng et al. (2020)
Osteoarthritis (OA)	Reverse collagen II and aggrecan degradation; Nrf2 and NF-κB signaling pathways (−)	MMP-13, ADAMTS-5, COX-2, iNOS, IL-6, TNF-α, PGE2↓	Luo et al. (2020)
	MAPK and Wnt/β-catenin signaling pathways (−)	MMP1, MMP13, ADAMTS-5, Wnt3a↓; COL2A1, ACAN↑	Liu et al. (2020b)
	TLR4/NF-κB pathways (−)	IL-1β, TNF-α, IL-6, SOD, MDA, ICAM-1, MCP-1, CASP3, Bax/Bcl-2, TLR4↓	Zhang et al. (2017)
	NF-κB and PI3K/AKT signaling pathways (−)	IL-1β, iNOS, MMP13, ADAMTS-5, COX-2, IL-6↓	Wang et al. (2019)
	NF-κB/MAPK signaling pathways (−)	MMP3, MMP9, MMP13, ADAMTS4, ADAMTS5↓	Yang et al. (2022)
	PI3K/AKT/mTOR signaling pathways (−)	COL2A1, SOX9, IL-6 APOA1, ABCA1↑; MMP13, CH25H, CYP7B1, AKT, p-AKT, mTOR, p-mTOR↓	Ju et al. (2021)
Colitis	pro-inflammatory cytokines (−); apoptosis and oxidative stress (−)	MDA, NO, IL-6, TNF-α, Bax↓; SOD, TAS, Bcl-2↑	Aksit et al. (2023)
Colitis-associated colorectal cancer	Hedgehog signaling pathway (−)	proliferation, migration, colony formation↓	Zeng et al. (2022)
Rheumatoid arthritis (RA)	Regulate the Keap1/Nrf2/HO-1 pathways	IgG, IgE, TNF-α, IL-1β, IL-6, Th1/Th2, HO-1↓; Keap1, Nrf2↑	Li et al. (2023a)
Cerebral ischemia/reperfusion damage	Regulate MAPK signaling Pathway	TNF-α, IL-1β, p-JNK, p-p38, iNOS↓; p-ERK1/2↑	Chen et al. (2020a)
	PI3K/Akt pathway (+)	Nrf2, HO-1, SOD↑; ROS, NF-κB↓	Xie et al. (2023)
	Inflammatory responses and oxidative stress (−); MAPK/NF-κB pathways (−)	MDA, SOD, GSH, IL-6, IL-1β, NO, p65, p38↓	Zhang et al. (2022)
Renal ischemia–reperfusion injury	MAPK pathway (−); Pro-inflammatory macrophage polarization (−)	ERK, p38, JNK↓	Deng et al. (2024)
Myocardial ischemia–reperfusion injury	NLRP3 inflammasome activation (−); Akt/mTORC1/NLRP3 signaling pathways (−)	LDH, CK-MB, Myo, ALT, IL-1β, IL-18, cas1, IL-1β, NLRP3, TNF-α↓	Xu et al. (2020)
Cognitive deficits	Alleviate LPS-induced cognitive disturbances	AChE, TNF-α, IL-6, Nrf2, LC3 II, mTOR, P62↓	Baluchnejadmojarad et al. (2018)
Neuroinflammation-astrocyte	TLR4/NF-κB pathways (−)	TNF-α, IL-1β, IL-6, iNOS↓; IL-4, BDNF↑	Lu et al. (2021)
Neuroinflammation-Macrophages	NLRP3 Inflammasome Activation in Macrophages (−)	CASP1, IL-1β↓	Liu et al. (2017b)
Neuroinflammation-microglia	Mitigate microglia inflammation; mir-7036a-5p/MAPT/PRKCG/ERK signaling pathways (−)	Mir-7036a-5p, TNF-α, IL-1β, iNOS↓	Duan et al. (2024)
	Neuroinflammation and microglia activation (−)	NLRP3, CASP1, IL-1β↓	Bian et al. (2020)
	Neuroinflammation (−); AKT/NF-κB and p38/JNK pathways (−)	TNF-α, NO, IL-6, IL-1β, iNOS↓	You et al. (2018)
Neuroinflammation-microglia, astrocyte	Microglia inflammatory activation (−)	NO, TNF-α, IL-1, ROS, iNOS, NF-κB, JNK, ERK↓	Wang et al. (2011)
Kidney injury	Inflammation and oxidative stress (−); regulate Nrf2/PPAR-c/PGC-1a/NF-κB/TLR4 pathways	BUN, ROS, MDA, IL-10, TLR4, NF-κB, TNF-α↓; Nrf2, HO-1↑	Shahmohammadi et al. (2023)
	NLRP3 inflammasome activation (−)	BUN, SCr, Kim-1, IL-18, NGAL, Cys-C↓	Li et al. (2020)
Asthma	Smad/MAPK pathways (−)	CDH1↑, CDH2, α-SMA↓	Li et al. (2024a)
Foam cell	Autophagy (+); NLRP3 inflammasome activation (−)	Plin2, LC3B-II, Map1lc3b, Becn1↑	Gao et al. (2024)
Intervertebral disc degeneration	Regulate Rab8a *via* the PI3K/PTEN/Akt pathway	p62↓, Beclin-1, Rab8a, ATG5↑	Hu et al. (2022)
	NLRP3 inflammasome activation (−); NF-κB/MAPK signaling pathways (−)	p-p38, p-JNK↓; p-ERK1/2↑	Wang et al. (2022b)

Annotation “+”: activate; “-”: suppress; “↑”: increase/upregulated; “↓”: reduce/downregulate.

### 4.1 Osteoarthritis

**FIGURE 6:**
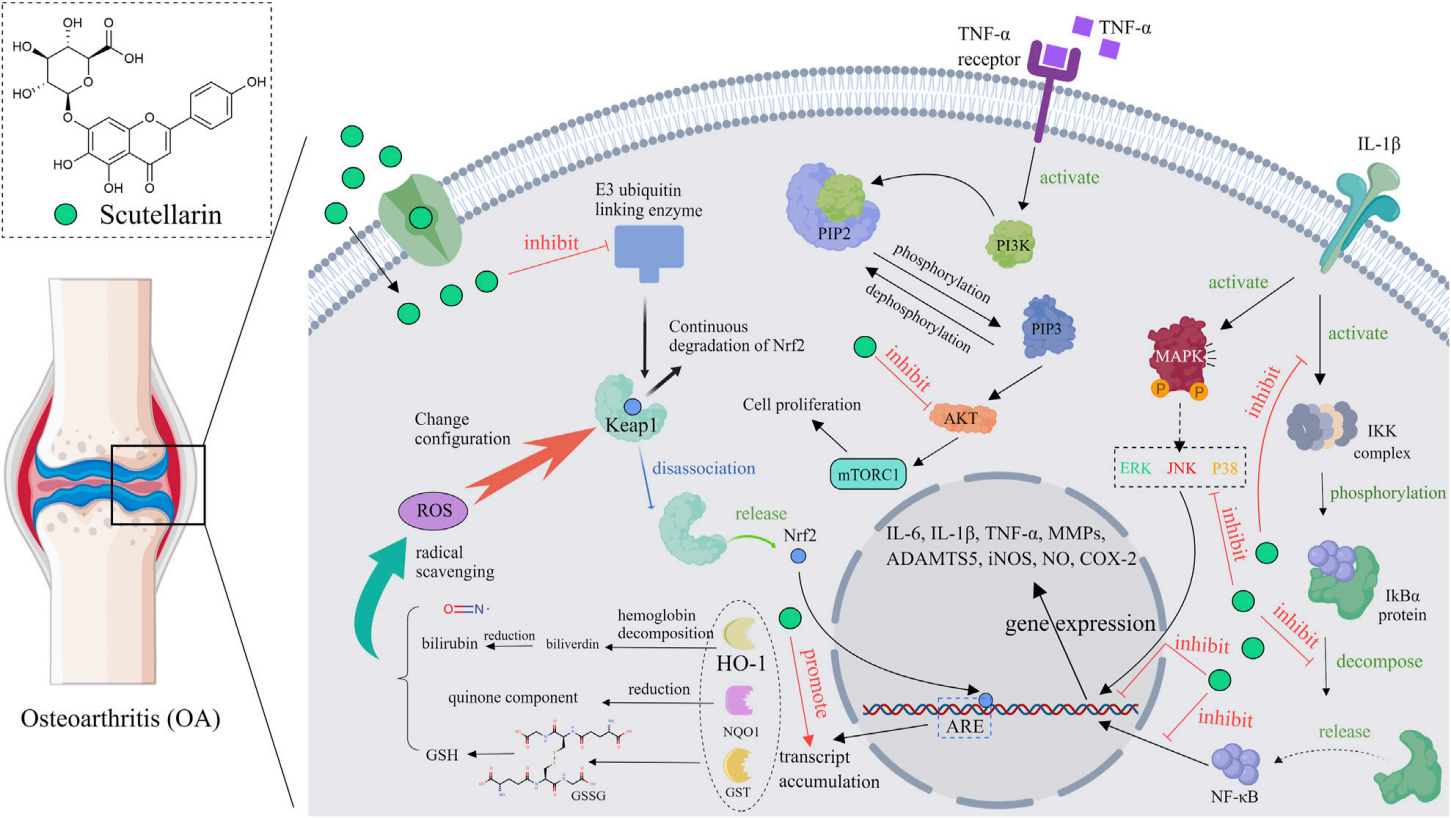
Diagram of the mechanism of action of scutellarin in the treatment of osteoarthritis, Created with MedPeer (medpeer.cn).

Osteoarthritis (OA), an age-related degenerative joint disease (Panieri and Saso, 2021), is characterized by the subchondral bone remodeling, progressive degradation of cartilage, chronic pain and synovitis (Ruan et al., 2023). OA is a complex joint disorder, inflammation and oxidative stress are the main etiologies of this disease (Zahan et al., 2020). Several *in vivo* and *in vitro* studies have discovered that scutellarin was effective in inhibiting the progression of OA. For example, scutellarin significantly reduced cartilage tissue loss and lowered osteoarthritis research society international (OARSI) score in destabilization of medial meniscus (DMM) model mice, showing good therapeutic potential (Yi et al., 2021). Mechanistically, scutellarin reduced the expression of inflammatory cytokines and the nuclear translocation of β-catenin through regulating NF-κB, MAPK (Yang et al., 2022), and Wnt/β-catenin pathways (Liu F. et al., 2020), thereby protecting the cartilage matrix from degradation. Moreover, scutellarin can slow down the progression of osteoarthritis by reducing inflammation-related protein expression in chondrocytes, which may be attributed to its inhibitory effect on the PI3K/AKT/mTOR pathway (Ju et al., 2021). Scutellarin can also enhance the expression of Nrf2 dependent HO-1 by binding to Nrf2, thus reducing inflammation and matrix degradation (Luo et al., 2020). As high cholesterol levels are the high risks of OA, scutellarin can reduce the progression of OA through regulating cholesterol metabolism related proteins (CH25H, CYP7B1, ABCA1, and APOA-1) (Choi et al., 2019), In summary, scutellarin has potential therapeutic value in the treatment of OA by inhibiting the NF-κB, MAPK, PI3K/AKT/mTOR, and Wnt/β-catenin pathways, as well as regulating the expression of cholesterol metabolism related proteins (Figure 6).

### 4.2 Pulmonary fibrosis

Pulmonary fibrosis is an irreversible lung disease characterized by collagen deposition in the alveolar spaces. Inflammatory response and oxidative stress are closely related to the pathogenesis of pulmonary fibrosis (Liu M. H. et al., 2017). Research has shown that scutellarin can alleviate the inflammatory response of pulmonary fibrosis by inhibiting the production of various inflammatory factors (Peng et al., 2020). Scutellarin has significant anti-pulmonary fibrosis effects by acting on the TGF-β/Smad and NF-κB signaling pathways (Liu et al., 2019) Furthermore, scutellarin can directly inhibit the activation, proliferation, and production of collagen in fibroblasts (Luo et al., 2020), thus slowing down the pathological process of pulmonary fibrosis (Liu et al., 2019; Ma et al., 2020). Scutellarin can also alleviate lung tissue damage caused by oxidative stress *via* activating the Nrf2/ARE signaling pathway, and increasing the expression of anti-oxidant related enzymes (SOD, CAT, and HO-1, etc.) (Hao et al., 2023). Some studies have shown that scutellarin can reduce the expression levels of fibronectin, MMPs, and TIMP (Peng et al., 2020), thereupon then prevent extracellular matrix (ECM) deposition and maintain lung structure (Zhou et al., 2019). In summary, scutellarin reduces ECM deposition by blocking the production of inflammatory factors, reducing oxidative stress, and inhibiting fibroblast proliferation, thereby playing a therapeutic role in pulmonary fibrosis. These mechanisms reveal the broad prospects of scutellarin in the treatment of pulmonary fibrosis.

### 4.3 Kidney injury

Kidney injury is a common clinical syndrome characterized by decreased renal function resulting from the accumulation of metabolic waste and toxins in the body (Li et al., 2022). Inflammatory response and oxidative stress play key roles in the pathogenesis of kidney injury (Andrianova et al., 2020). Scutellarin inhibits the production of pro-inflammatory cytokines by suppressing the NF-κB signaling pathway, thereby alleviating inflammatory damage to renal tissue (Liu et al., 2022). Scutellarin can also increase the expression of anti-oxidant enzymes (SOD, CAT, HO-1, etc.) by activating the Nrf2/ARE signaling pathway, thereby protecting kidney tissue from oxidative stress damage (Shahmohammadi et al., 2023). In addition, scutellarin protects renal function by inhibiting the PI3K/Akt pathway and reducing apoptosis of renal tubular epithelial cells (TECs) (Liu et al., 2022). TGF-β and Smad proteins can activate the production of fibrosis markers (collagen, fibronectin, etc.), while scutellarin can precisely inhibit the expression of TGF-β and Smad, and then reduced renal fibrosis (Wang H. et al., 2022). In summary, the effects of scutellarin on renal injury may be mediated through multiple pathways, such as inhibiting inflammation, oxidative stress, apoptosis of renal tubular epithelial cells, and renal fibrosis.

### 4.4 Cardiovascular diseases

Cardiovascular diseases (CVDs) are the leading cause of death and disability worldwide, closely associated with oxidative stress and inflammatory responses (Zhong et al., 2019). Scutellarin has anti-inflammatory and anti-oxidant properties, which can reduce the occurrence of CVDs. Scutellarin can inhibit the MAPK and NF-κB signaling pathways, thus reducing the inflammatory response caused by cardiovascular injury models (Zhang et al., 2022). Scutellarin can also reduce ROS production by activating the Nrf2/ARE signaling pathway, further alleviated oxidative stress-induced cardiovascular damage (Wang and Ma, 2018; Zhang et al., 2023). In addition, scutellarin can reduce the accumulation of lipids and the formation of foam cells (Gao et al., 2024), thereby reducing the generation of atherosclerosis. Remarkably, scutellarin can stabilize atherosclerotic plaque, reduce the risk of plaque rupture, and limit the occurrence of subsequent cardiovascular events (Xu et al., 2021). In summary, scutellarin has preventive and therapeutic effects on cardiovascular diseases by inhibiting the production of inflammatory factors, reducing oxidative stress levels, and stabilizing plaques.

## 5 Pharmacokinetic characterization of scutellarin and dosage form modification studies

Although scutellarin exerted strong anti-inflammatory activity in many cellular experiments, *in vivo* pharmacokinetic studies have indicated that scutellarin have some shortcomings for the treatment effects. The absolute oral bioavailability of scutellarin in Beagle dogs was reported to be 0.40% ± 0.19% (Ge et al., 2003), which was hardly absorbed. Scutellarin is also rapidly metabolized and excreted after intravenous injection, with a short elimination half-life of (52 ± 29) min (Jiang et al., 2003; Wu et al., 2021). Moreover, the chemical structure of scutellarin has phenolic hydroxyl group, it will be easy to produce precipitation in acidic solution. Studies have shown that flocculent precipitation could occur when scutellarin is dissolved in the infusion solution with pH < 3.8 (Xu et al., 2012), which implies that its absorption is reduced in gastrointestinal tract, partly explaining the low bioavailability.

**FIGURE 7:**
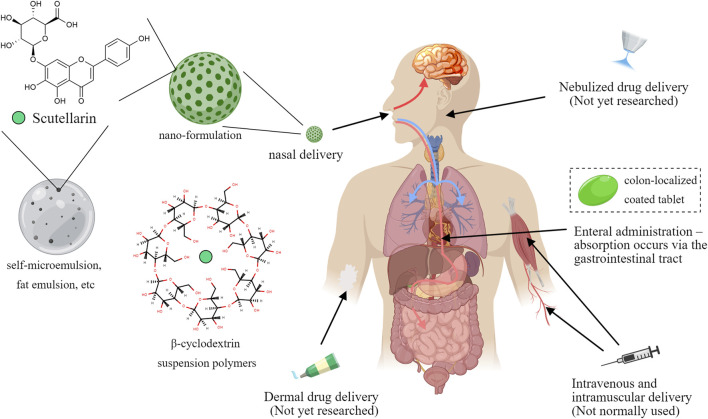
Forms and modes of administration of scutellarin, Created with MedPeer (medpeer.cn).

To address the problem, more and more modern technologies have been gradually applied to improve the effectiveness of scutellarin. On the one hand, various special materials are used to improve the dosage form. For example, β-cyclodextrin suspension polymers are used as carriers to enhance the solubility of scutellarin (Liao et al., 2020); the triglyceride mimetic prodrug of scutellarin was designed to promote intestinal lymphatic transport and improve oral bioavailability (Wang et al., 2021); liposome precursors of scutellarin were also prepared using liposome technology, which improved the stability of scutellarin *in vivo* and enhanced its bioavailability (Luo et al., 2019). Furthermore, other new dosage forms, such as encapsulation technology, self-microemulsion, and fat emulsion, have also been widely used in the study of scutellarin formulations, which have improved the bioavailability, solubility and safety of scutellarin (Tian et al., 2014). On the other hand, the drug targeting of scutellarin was also explored in various ways. For cerebrovascular diseases, the blood-brain barrier was a major pain point hindering drug action, for which administration *via* the nasal route has emerged (Liu and Ho, 2017). *In situ* gels based on nanosuspensions have been developed as intranasal delivery formulations of scutellarin and have been shown to improve its solubility and bioavailability, as well as prolong its retention time in the nasal cavity (Chen Y. et al., 2020). PLGA-PEG-AEAA nano-formulations of scutellarin have been developed to promote tumor delivery (Li L. et al., 2023). Besides, the traditional coating technology was also introduced to increase drug targeting. Scutellarin could be prepared as a colon-localized coated tablet, and the drug would be released on the colonic site at pH > 7.0, which significantly promoted the colon-targeted drug delivery (Li et al., 2013) (Figure 7).

## 6 Discussion

As a natural flavonoid, scutellarin possesses significant anti-inflammatory properties, and has been identified as a promising candidate drug for the treatment of multiple inflammation-related diseases. According to the existing publications, the anti-inflammatory effects of scutellarin could be attributed to acting on several key inflammation signaling pathways such as MAPK, NF-κB, and PI3K/Akt, which reduced the production of pro-inflammatory mediators and consequently attenuated inflammation (Chen H. L. et al., 2020; Liu F. et al., 2020). Scutellarin can inhibit the activation of NF-κB, thereby reducing the expression of TNF-α and IL-1β (Ju et al., 2021; Aksit et al., 2023; Li J. et al., 2023). In addition, scutellarin can exert anti-oxidant effects by triggering the Nrf2/ARE signaling pathway and increasing the expression of anti-oxidant enzymes, thus reducing cell damage caused by oxidative stress (Xie et al., 2023). These results indicated that scutellarin have potential application value in the prevention and treatment of various inflammation-related diseases, such as osteoarthritis (Wang et al., 2019; Liu F. et al., 2020; Zahan et al., 2020; Yang et al., 2022), pulmonary fibrosis (Hao et al., 2023), ischemia/reperfusion injury (Xie et al., 2023), cisplatin induced acute renal injury (Shahmohammadi et al., 2023), and diabetes induced cardiomyopathy (Xu et al., 2021).

According to the latest research results, the clinical application prospects of scutellarin are very broad. Scutellarin is a naturally extracted metabolite with good safety (Sun et al., 2019; Zhang et al., 2020), making it an ideal molecule for clinical applications. The multi-target ability of scutellarin makes it an anti-inflammatory drug with a wide range of pharmacological effects on complex inflammatory diseases (Peng et al., 2020). Researchers are currently researching new dosage form technologies to overcome the drawbacks caused by the properties of scutellarin, such as increasing the solubility and dissolution rate of scutellarin. These new dosage forms could not only increase the bioavailability, but also improve medication safety. However, there are still some problems in the dosage form studies, such as repetitive experiments, *in vivo* pharmacokinetics and drug release behavior studies. Hence, it is necessary to carry out in-depth studies on the *in vivo* pharmacokinetics and biopharmacy of the new dosage form. Moreover, the opening of new routes of drug delivery (transdermal, mucosal, nebulization, etc.) may bring new perspective to the drug development of scutellarin. Precision therapy should also be incorporated in the pre-clinical investigation of scutellarin using genomics and proteomics technologies.

In conclusion, scutellarin, as a typical flavonoid with multiple pharmacological effects and low toxicity, can be applied in anti-inflammatory, anti-oxidant and antitumor fields in the future. It is believed that scutellarin will play a prominent role in the treatment of various inflammatory diseases and provide new therapeutic options for patients through optimized formulations, combination therapies and in-depth studies on its mechanism.

## Figures and Tables

**Table:** 

**AChE activity**	acetylcholinesterase
**ADAMTS-5**	a disintegrin and metalloproteinase with thrombospondin motifs 5
**Akt**	protein kinase B
**ARE**	anti-oxidant response element
**Bax**	Bcl-2 associated X protein
**BUN**	blood urea nitrogen
**CAT**	catalase
**COX-2**	cyclooxygenase-2
**ERK**	extracellular signal regulated kinase
**HO-1**	heme oxygenase-1
**IKK**	IκB kinase
**iNOS**	inducible nitric oxide synthase
**IL-1**	interleukin-1
**IL-1β**	interleukin-1β
**IL-6**	interleukin 6
**JNK**	Jun N-terminal kinase
**Keap1**	kelch-like ECH-associated protein 1
**MAPK**	mitogen-activated protein kinase
**MAPKK**	MAPK kinase
**MAPKKK**	MAPKK kinase
**MDA**	malondialdehyde
**MMP-13**	matrix metalloproteinase 13
**MMP-2**	matrix metalloproteinase 2
**mTORC2**	mechanistic target of rapamycin complex 2
**NO**	nitric oxide
**Nrf2**	nuclear factor erythroid 2-related factor 2
**NF-κB**	nuclear factor-kappa B
**PDK1**	pyruvate dehydrogenase kinase, isozyme 1
**PGE2**	prostaglandin E2
**PI3K**	phosphatidylinositol3 kinase
**PIP2**	phosphatidylinositol 4,5-bisphosphate
**PTEN**	phosphate and tension homology deleted on chromosome ten
**ROS**	reactive oxygen species
**Smad**	*drosophila* mothers against decapentaplegic protein
**SOD**	superoxide dismutase
**TAS**	total anti-oxidant status
**TGF-β**	transforming growth factor-β
**TLR4**	toll-like receptor 4
**TNF-α**	tumor necrosis factor-α
